# Capturing multi-stage fuzzy uncertainties in hybrid system dynamics and agent-based models for enhancing policy implementation in health systems research

**DOI:** 10.1371/journal.pone.0194687

**Published:** 2018-04-25

**Authors:** Shiyong Liu, Konstantinos P. Triantis, Li Zhao, Youfa Wang

**Affiliations:** 1 Research Institute of Economics and Management, Southwestern University of Finance and Economics, Chengdu, Sichuan, China; 2 Grado Department of Industrial and Systems Engineering, Virginia Polytechnic Institute and State University, Falls Church, VA, United States of America; 3 Department of Health Policy and Health Management, West China School of Public Health, Sichuan University, Chengdu, China; 4 Fisher Institute of Health and Well-Being, Ball State University, Muncie, IN, United States of America; Southwest University, CHINA

## Abstract

**Background:**

In practical research, it was found that most people made health-related decisions not based on numerical data but on perceptions. Examples include the perceptions and their corresponding linguistic values of health risks such as, smoking, syringe sharing, eating energy-dense food, drinking sugar-sweetened beverages etc. For the sake of understanding the mechanisms that affect the implementations of health-related interventions, we employ fuzzy variables to quantify linguistic variable in healthcare modeling where we employ an integrated system dynamics and agent-based model.

**Methodology:**

In a nonlinear causal-driven simulation environment driven by feedback loops, we mathematically demonstrate how interventions at an aggregate level affect the dynamics of linguistic variables that are captured by fuzzy agents and how interactions among fuzzy agents, at the same time, affect the formation of different clusters(groups) that are targeted by specific interventions.

**Results:**

In this paper, we provide an innovative framework to capture multi-stage fuzzy uncertainties manifested among interacting heterogeneous agents (individuals) and intervention decisions that affect homogeneous agents (groups of individuals) in a hybrid model that combines an agent-based simulation model (ABM) and a system dynamics models (SDM). Having built the platform to incorporate high-dimension data in a hybrid ABM/SDM model, this paper demonstrates how one can obtain the state variable behaviors in the SDM and the corresponding values of linguistic variables in the ABM.

**Conclusions:**

This research provides a way to incorporate high-dimension data in a hybrid ABM/SDM model. This research not only enriches the application of fuzzy set theory by capturing the dynamics of variables associated with interacting fuzzy agents that lead to aggregate behaviors but also informs implementation research by enabling the incorporation of linguistic variables at both individual and institutional levels, which makes unstructured linguistic data meaningful and quantifiable in a simulation environment. This research can help practitioners and decision makers to gain better understanding on the dynamics and complexities of precision intervention in healthcare. It can aid the improvement of the optimal allocation of resources for targeted group (s) and the achievement of maximum utility. As this technology becomes more mature, one can design policy flight simulators by which policy/intervention designers can test a variety of assumptions when they evaluate different alternatives interventions.

## Introduction: Background and motivation

**Fig 1:**
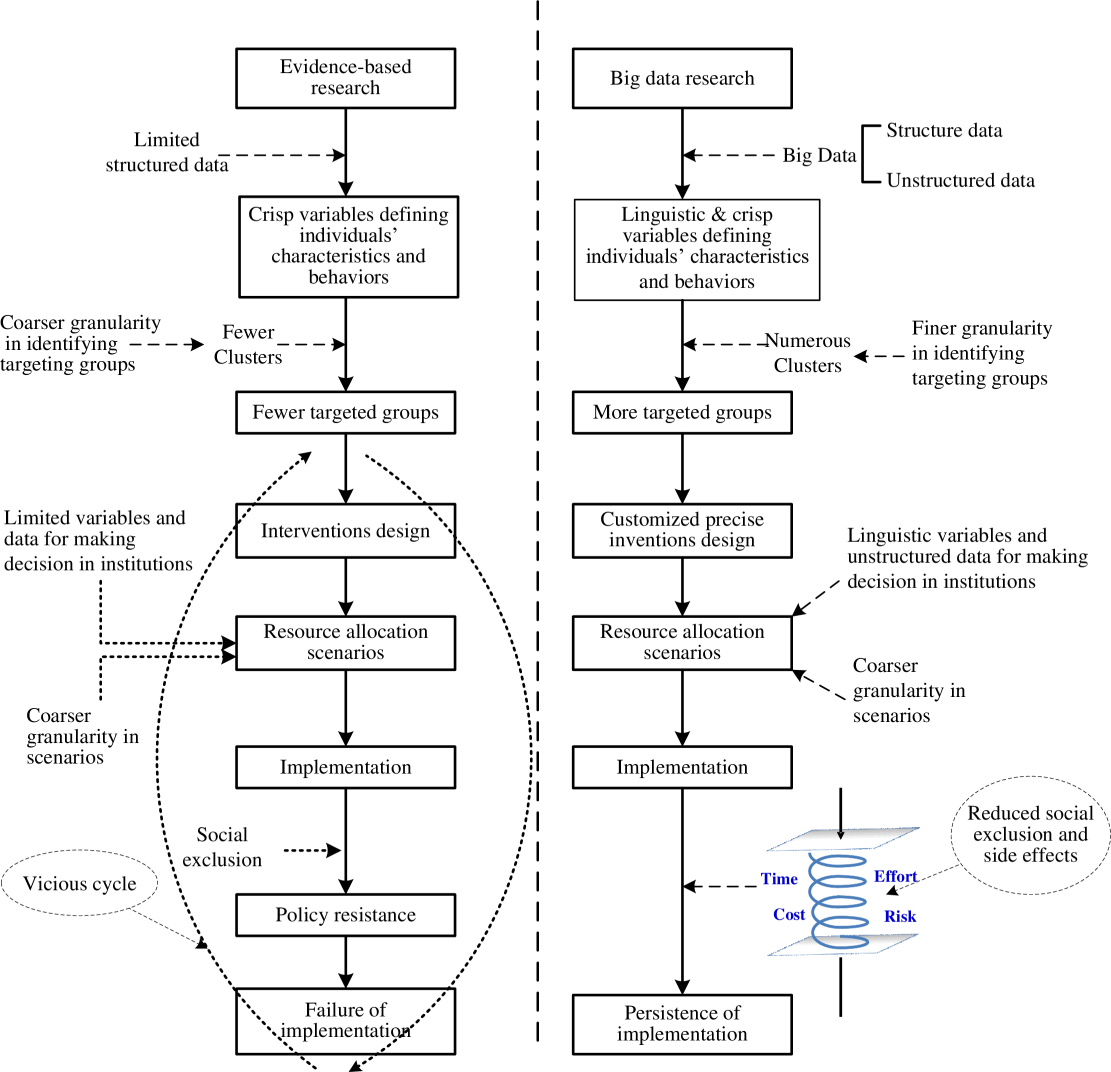
Multi-stage uncertainties manifested in stakeholder decision-making processes during implementation.

Implementation research has become popular since the beginning of the 21st century. It translates research findings into sustainable interventions [1]. Policy design and the implementation of interventions were traditionally based on evidence-based research (with limited data) along with the feedback from implementation efforts. When using this approach, the obtained "evidence" is typically not sufficient to fully implement a designed policy that leads to health interventions. It has to go through the three steps, i.e., principle adoption, early implementation, and implementation persistence [2]. However, there are multi-stage uncertainties in stakeholder decision making during implementation (Fig 1).

However, data obtained from different sources of socioeconomic systems, especially at the individual level, contain enormous amounts of unstructured data that depict people's decision-making process and consequently their behaviors [3, 4]. It was found that most people made health-related decisions not based on numerical data but based on perceptions [5–8]. Examples include the perceptions and their corresponding linguistic values of health risks such as, smoking, syringe sharing, eating energy-dense food, drinking sugar-sweetened beverages etc. Therefore, to better understand the mechanisms that affect the continued implementation of health interventions, we employ fuzzy variables to quantify linguistic variables in healthcare modeling [9–12].

In traditional evidence-based research (e.g., randomized clinical trial-RCT) and the ensuing modeling processes, structured data allow researchers and policy makers to define individuals' perceptions and behaviors using crisp variables. These crisp variables result in coarser granularity when identifying target intervention groups. The design of relevant interventions might ignore the needs of individuals or small groups that cannot be identified in the large groups that are defined under the coarse-granularity screening. In this case, the policy implementation can cause social exclusion (where people are worse off as their needs are not identified and addressed) and policy resistance. With the help of linguistic variables that portray individual's characteristics and behaviors, researchers and policy makers can identify groups (clusters) and their needs with the finer-granularity screening. Therefore, customized precise interventions can be implemented on targeted groups [1, 2] (refer to Fig 1).

In the domain of policy implementation research, two simulation approaches, i.e., system dynamics modeling (SD) and agent-based modeling (ABM) are widely employed to better understand the structure of complicated socioeconomic systems and the impact of relevant policies. The major differences between the two simulation approaches are the assumptions on which they are based. ABM focuses on evolution and heterogeneity of interacting agents, and how certain interventions can influence the characteristics, preferences, and behaviors of individuals. No consistent mathematical formalism characterizes ABMs [13]. SD models, on the contrary, have their foundation in ordinary differential equations. A very important feature of the SD approach is that the state variables (stock aggregating homogeneous agents) and flow (to and from) the stock are homogenous. SD is generally used to understand the how decisions or policies such as resource allocations might impact system structure and consequently the behaviors of key variables characterizing the system [14].

During the past two decades, more scholars started to explore the integration of SDM and ABM [15, 16]. Applications in different field have been addressed such as transportation [17], health-related research [18–21], psychology [22], work environment [23], ecological modeling [24–27]. In order to simultaneously capture characteristics of heterogeneity and homogeneity in modeling complex socioeconomic systems in dynamic simulation environment, two types of hybrid models that combine ABM and SD were explored. In the first type, ABM is used to create the aggregate construct and then SD uses the aggregate structure to generate dynamic behaviors. Specifically, dynamic agents (automata), under given rules, interact with each other in a random manner and change their characteristics (or utility) under the influence of peer agents. As a result, agents with similar features or behaviors will aggregate into a cluster. This constitutes a phase transition from heterogeneity to homogeneity. Based on the cluster characteristics, resource allocation strategies for different interventions are initiated on the homogeneous clusters and system dynamic behaviors are hence explored. As for the second type hybrid model, SD is used at the microscopic level to derive the features (state) of agents (agents could be institutions, processes, etc.) and ABM deals with the interactions of those agents under different rules [28]. In this research, we mainly address fuzzy variables (using them to process unstructured data), their operations, and algorithms with the first type of hybrid model.

Zimmermann stated that: “*Uncertainty implies that in a certain situation a person does not dispose about information which quantitatively and qualitatively is appropriate to describe*, *prescribe or predict deterministically and numerically a system*, *its behavior or other characteristics*” ([29], p. 192). In order to better exploring and understand the complex socioeconomic systems and dynamics of action-reaction of human interventions, many uncertainties have to be captured and manifested. Many theories have been proposed to enhance the modeling of uncertainties. Hartley [30] employed class set theory to explore the measurement of uncertainty. Probability theory was used by Shannon [31] to measure uncertainty. Zadeh, in 1965, proposed fuzzy set theory [32–35] to examine the uncertainties related to using linguistic variables in decision-making process. Shafer [36] used evidence theory and Dubois and Prade [37] employed possibility theory to investigate the measurement and modeling of uncertainties. Apparently, fuzzy set theory has been widely recognized and used given the benefits of representing multiple linguistic values associated with a specific variable or phenomenon. One of the basic premises of fuzzy set theory is that it can represent the degree of truth or relevance of a specific phenomenon. The basic definition for fuzzy set is as follows.

Suppose that U is the universe of discourse, for each *x* ∈ U, the function *μ* = *f*(*x*) ∈ [0,1] holds, the value *μ* is called the degree of membership of *x* in set A = {(x,*μ*) | x ∈ U, *μ* = f(x), f ⊆ U × [0,1]}, *A* is called fuzzy set and *μ* = f(x) is called the membership function of fuzzy set *A*. If *x* ∈ *U*, *x* is not included in fuzzy set *A* if *μ* = 0; *x* is fully included if *μ* = 1; *x* is partially included (a degree of membership) in the fuzzy set *A* [32–35].

Thus far, some research has been conducted to apply fuzzy logic to SD models to explore the use of linguistic variables in resource allocations, institution decisions, and policy evaluation [38–41]. Few applications of fuzzy logic to ABM were also attempted in the literature [42, 43]. Nevertheless, no research has been conducted that incorporates fuzzy logic especially using high-dimensional data in a hybrid model that combines SD and ABM, let alone providing a mathematical framework. The objective of this research is to provide the mathematical formalism for incorporating fuzzy variables at multiple stages of the first type of a hybrid AGM and SD model discussed earlier that includes interacting fuzzy agents as well as aggregate level resource allocations. The mathematical formulations for 5 types of dynamics are exhibited: 1) the agents characterized by linguistic variables are able to transition from being heterogeneous to homogeneous [44]; 2) differing from a standalone ABM where interventions are preset, the interventions and their impacts in this model are emergent and are affected by the dynamics of the resource allocations that are addressed by the SD model; 3) once fuzzy agents form certain clusters, they are continuously affected by the dynamic allocations until an optimal status is achieved; 4) the formation of clusters of these fuzzy agents also conversely affects the resource allocation dynamics by feeding information back to the SD model part; 5) in the nonlinear-causal loop, the institutional decision-making process that incorporates fuzzy variables is pivotal in driving the dynamics of whole hybrid model. This helps inform and advance implementation research and helps achieve the sustainability of policies and the persistence of implementations [45–47].

This research provides three major contributions to the literature and implementation practice of health policies. First of all, it systematically exhibits the way of integrating ABM and SD models in a single hybrid simulation model which, for purpose of optimizing resource allocation in health interventions, helps to simultaneously capture the homogeneity of clustered (segmented) populations at aggregate level and the heterogeneity of agents at individual level. Secondly, it incorporates unstructured data that portray the perceptions, preferences, behaviors, and decisions of individuals into the hybrid simulation model. Last but not the least, this research intends to employ fuzzy set theory to portray linguistic variables that represent individuals' decisions and behaviors. It also describes the way of using high dimensional data in a hybrid simulation model where an individual's decisions and behaviors resulted from dynamics of multiples linguistic variables and interactions.

This paper is organized as follows. In Section 1, we present the background and motivation for conducting this research. Section 2 depicts how linguistic variables are represented as part of interacting agents that are affected by the dynamics of the interventions that are generated from resource allocation decisions. Section 3 depicts how linguistic variables are represented in system dynamics models. The last section concludes this research by discussing issues, challenges, and future research in terms of theory and applications.

## Methodology

### Interacting, clustering, and decision-making of fuzzy agents

#### Definition of fuzzy agents in a simulation environment

In a simulation environment for a hybrid model that combines ABM and SD, *A* is a set of agents (e.g., interacting individuals) denoted by *a*_*i*_ ∈ SA, i ∈ *N*, where *SA* = {*sa*_1_,…*sa*_*i*_,…,*sa*_*n*_},; based on Zadeh's quintuple definition of linguistic (fuzzy) variables, i.e., $\left(X,T,U,G,\tilde{M}\right)$, U is the universe of discourse U = {x1, x2,…,xn}, *X*, for example, is perceived "temperature", *T* is the set of T = {Low,Medium,High} defined by *G* which is the syntactic rule for generating linguistic terms, $\tilde{M}$ represents the semantic rule of associating a linguistic term with its meaning.

**Fig 2:**
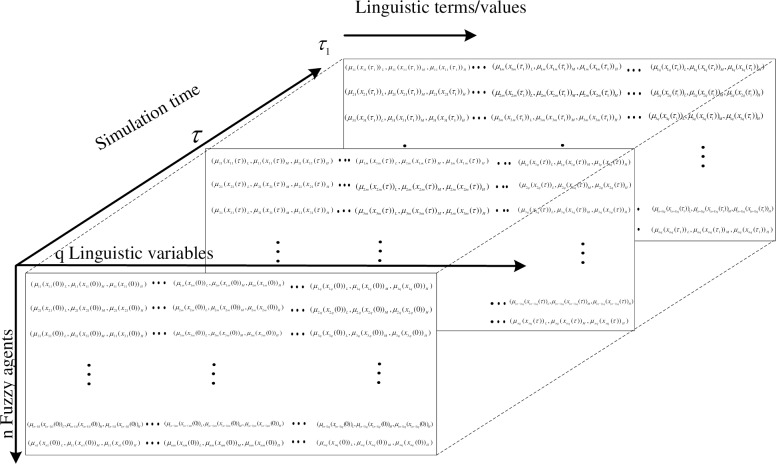
Representation of change over time of high-dimensional *n* fuzzy agents having *q* linguistic variables.

In this research, let the set $A=\{(X_{1},T_{1},U_{1},G_{1},\;\tilde{M_{1}})\ldots ,(X_{q},T_{q},U_{q},G_{q},\tilde{M_{q}})\}$ be the set of *q* linguistic variables [32–35] characterizing the perceptions of given agents. Here, U = {*U*_1_, *U*_2_,…,*U*_3_} and U_j_ = {x_j1_, x_j2_,…,x_jn_}, X_j_ is the *j*^*th*^ linguistic variable characterizing the *i*^*th*^ agent, where *q* ≤ *j* ∈ N, *q* is the number of linguistic variables, *T*_j_ = {Low,Medium,High} (*T*_j_ is the set of linguistic terms defining the *j*^*th*^ linguistic variable) ranging over a universe of discourse *U* associated with the linguistic variable *x*_*j*_(*t*), where *x*_*j*_(*t*) is the *j*^*th*^ state variable changing over time in the SD simulation environment. For different linguistic variable, the linguistics term/value can be different. For example, if the chosen linguistic variable is "age", the linguistic terms here {young, middle-aged, old}. In the simulation environment, *x*_*j*_(*t*) represents the value of x(*t*) at time t of the j^th^ state variable. In some cases, two variables are used in SD simulation environment to accomplish the representation of linguistic variable with one as base variable, i.e., the normal state variable in model and perceived value of base variable. This means *x*_*j*_(*t*) is a function of time in the given context, where *x*_*j*_(*t*) ∈ *U*_*j*_, *U*_*j*_ = {*x*_*j*1_(*t*),…,*x*_*ji*_(*t*),…,*x*_*jn*_(*t*)}, *j*,*i* ∈ N, *U*_*j*_ stands for the set of values over time that the *j*^*th*^ state variable can take; among which, *x*_*j*_(*t*) ∈ *U*_*j*_, *μ*_j_: *U*_*j*_ → [0,1] is the membership function of *A* and *μ*_j_(*x*_*j*_(*t*)) ∈ [0,1] is the degree of membership/belongingness of *x*_*j*_(*t*) in A. And in this definition, the membership function for each linguistic term of the linguistic variable of particular agent are defined as follows, where for example, *μ*_ij_(x_ij_(*t*))_*Low*_ is the "Low" term of *j*^*th*^ linguistic variable of *i*^*th*^ agent in the simulation environment (referring to Eqs 1–3 and Fig 2).

$$\mu_{ij}{\left(x_{ij}(t)\right)}_{Low}=\left\{ \begin{array}{ll} \;1 & \;if\;x_{ij}(t)\in [0,b_{ij}],b_{ij}>0\\ \frac{c_{ij}-x_{ij}(t)}{c_{ij}-b_{ij}} & if\;x_{ij}(t)\in [b_{ij},c_{ij}],0<b_{ij}<c_{ij}\\ \;0 & \;other \end{array}\right.$$ (1)

$$\mu_{ij}{\left(x_{ij}(t)\right)}_{Medium}=\left\{ \begin{array}{ll} \frac{x_{ij}(t)-b_{ij}}{c_{ij}-b_{ij}} & if\;x_{ij}(t)\in [b_{ij},c_{ij}],\;0<b_{ij}<c_{ij}\\ \frac{d_{ij}-x_{ij}(t)}{d_{ij}-c_{ij}} & if\;x_{ij}(t)\in [c_{ij},d_{ij}],0<c_{ij}<d_{ij}\\ \;0 & \;other \end{array}\right.$$ (2)

$$\mu_{ij}{\left(x_{ij}(t)\right)}_{High}=\left\{ \begin{array}{ll} \frac{x_{ij}(t)-c_{ij}}{d_{ij}-c_{ij}} & if\;x_{ij}(t)\in [c_{ij},d_{ij}],0<c_{ij}<d_{ij}\\ \;1 & if\;x_{ij}(t)\in [d_{ij},+\infty ],d_{ij}>0\\ \;0 & \;other \end{array}\right.$$ (3)

**Fig 3:**
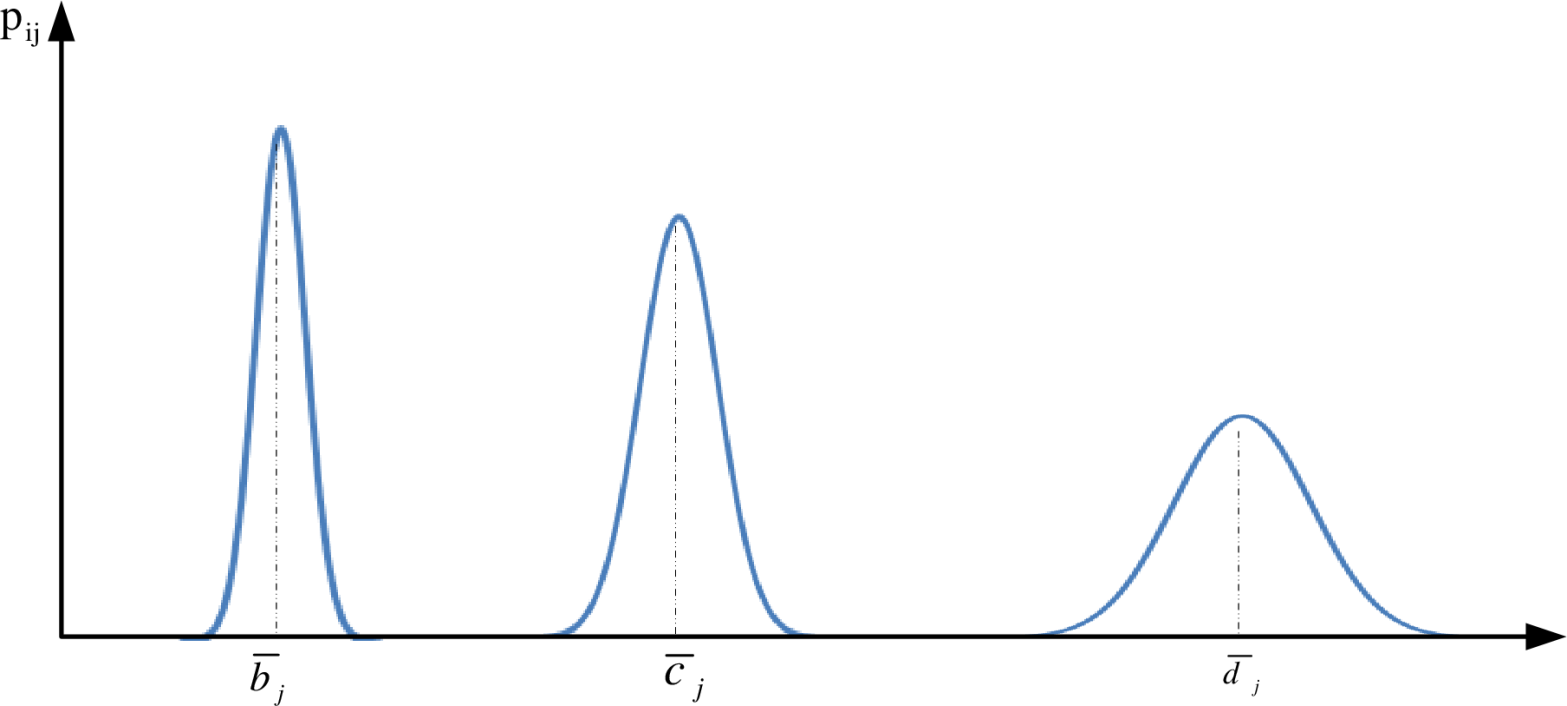
Normal distribution for the constants defining membership functions of *j*^*th*^ linguistic variable of n agents.

For the purpose of simplicity and to illustrate the concept, all the membership functions in this paper are triangular membership functions with three linguistic terms i.e. Low, Medium, and High. The framework can be easily generalized to use other types of fuzzy membership functions (e.g. trapezoidal, bell-shaped membership functions, or even more complex membership functions). The constants b_ij_, c_ij_, and *d*_ij_ denote the constants defining the triangular membership functions for *j*^*th*^ linguistic variable characterizing *i*^*th*^ agent in the simulation environment, which are assumed to follow normal distribution X∼N(*μ*,σ^2^) denoted by $B_{j}∼N(\bar{b_{j}},\sigma_{jb}^{2})$, $C_{j}∼N(\bar{c_{j}},\sigma_{jc}^{2})$, $D_{j}∼N(\bar{d_{j}},\sigma_{jd}^{2})$ respectively(refer to Fig 3).

**Fig 4:**
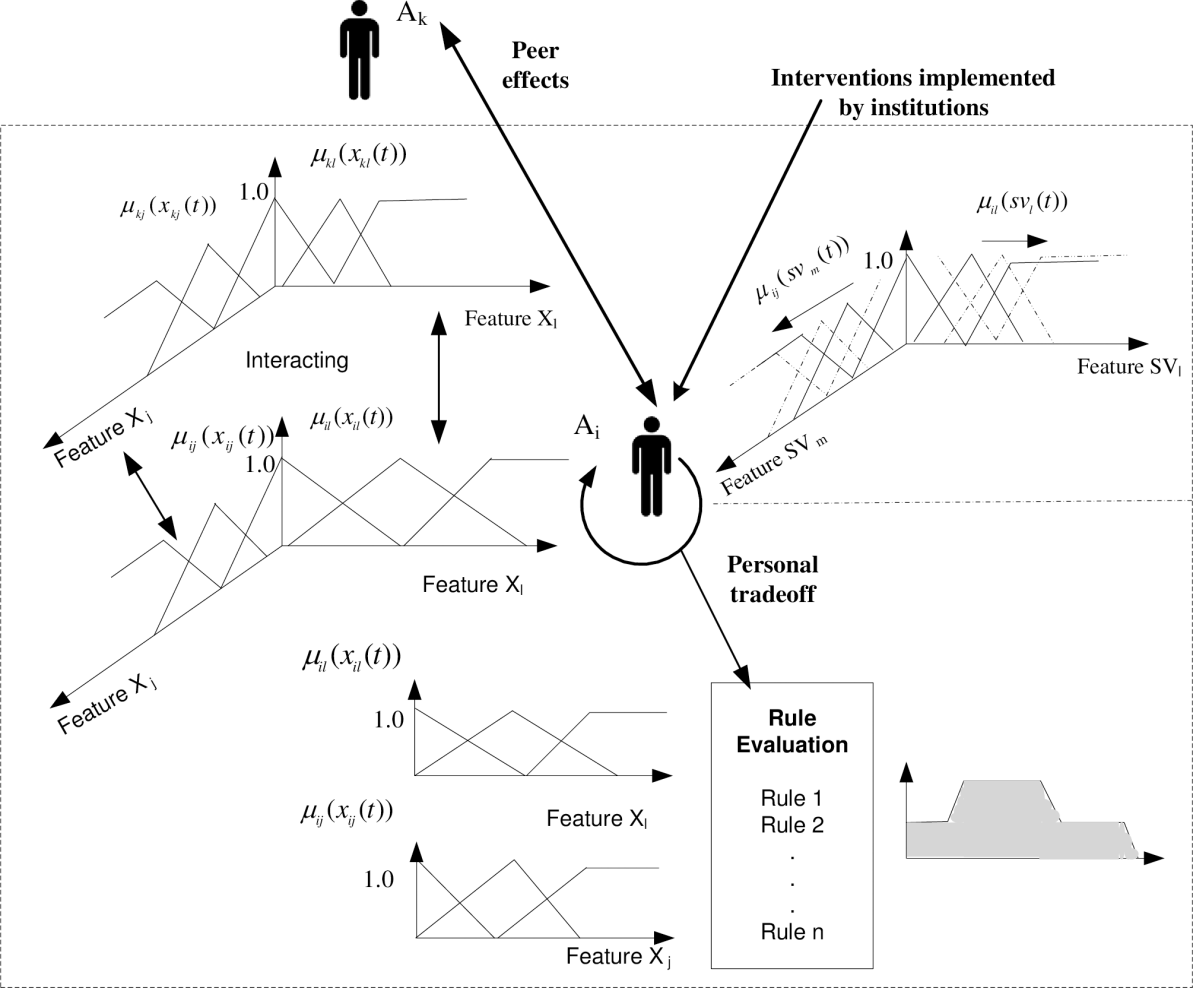
Portrayal of the dynamics of fuzzy agents acting based on personal decisions, institutional interventions, and impacts of other peer fuzzy agents.

There are three types of dynamics generated by the interventions in the hybrid simulation environment. Impacts exerted by interventions on individuals in the target population generate one type of dynamics. The values of linguistic terms (Low, Medium, and High) of *j*^*th*^ linguistic variable of *i*^*th*^ agent are determined by the value *x*_*ij*_(*t*) obtained at a certain time period, which is one of the variables that characterize the outcomes of the interventions implemented by institutions (top right of Fig 4). Peer effects driving agents to change their preference and status create another type of dynamics (top left of Fig 4). The third type of dynamics comes from the decision-making process where the agent incorporates the linguistic variables (*sv*_*l*_(*t*) i.e., the capacity of stores serving healthy food in a certain community, *sv*_*m*_(*t*) the capacity of outdoor activity spaces, etc.) and aggregating the linguistic variables that characterize an agent [40]. However, from the perspective of precisely targeted intervention, it is not meaningful to cluster agents with respect to different linguistic variables in all subspaces. The third type of dynamics is mainly examined at the institutional level decision-making that initiates interventions, which are depicted in the SD model in third part of this paper.

For sake of brevity, in the simulation algorithm, we assume that the generation of the above three types of dynamics follows the order that we introduce them in.

#### Impact of interventions on agents—first dynamic behavior

In the simulation environment, since the linguistic variable does not necessarily change linearly as defined in the standard definition, the values of the linguistic terms do not exhibit the standard triangular shape. Two examples are provided to demonstrate how linear and nonlinear changes over time affect the behavior of linguistic variables over time.

**Fig 5:**
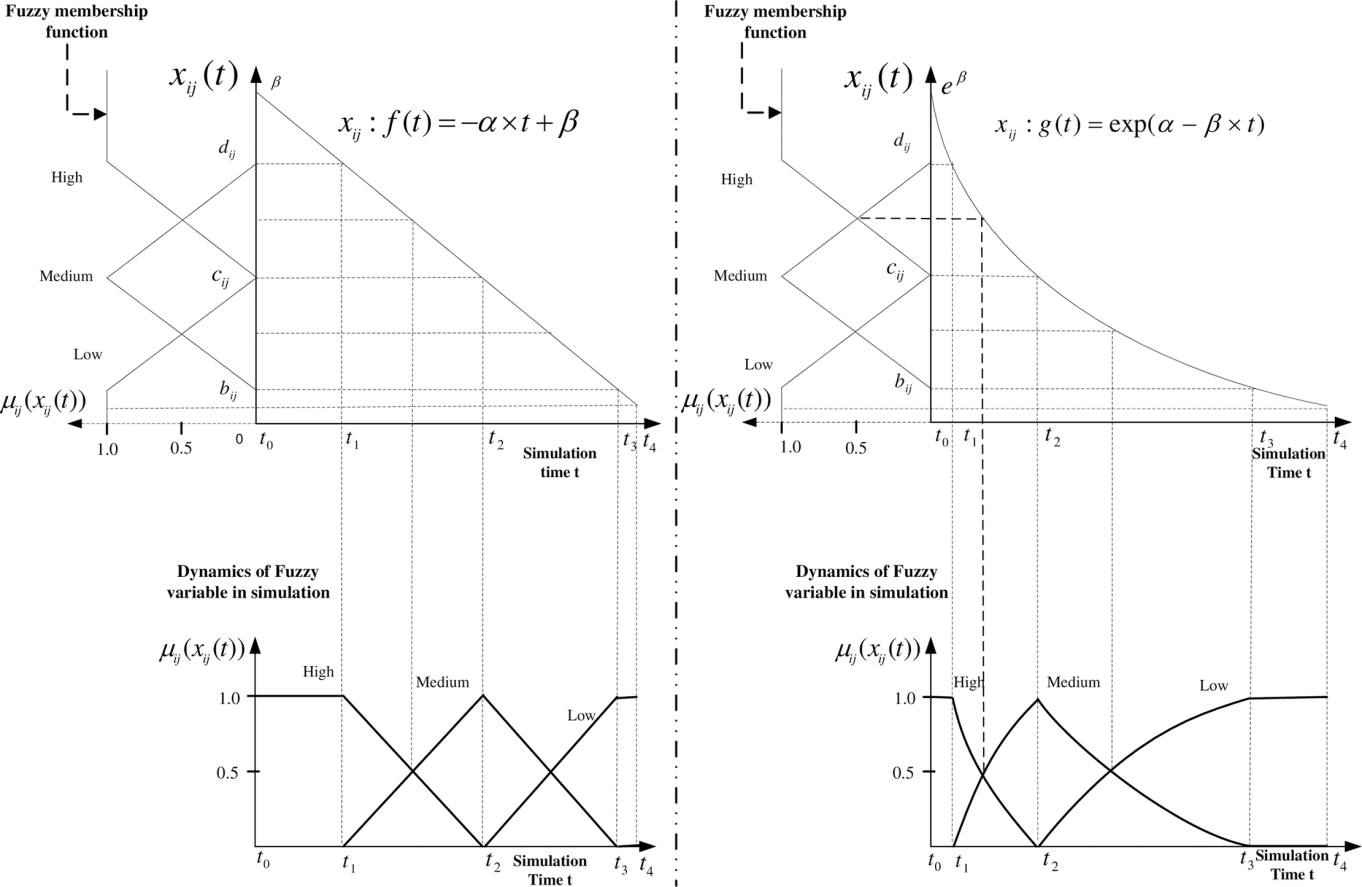
Impacts of interventions on agents.

Example 1: Suppose the change of *x*_*ij*_ over time is characterized by the function of *x*_*ij*_: *f*(*t*) = −*α* * *t* + *β* (upper left of Figs 4 and 5) where *α*,*β* > 0, the time horizon for the simulation is t_4_. We substitute *x*_*ij*_(*t*) with −*α* * *t* + *β*. The corresponding membership functions over time are:

$$\mu_{ij}{\left(x_{ij}(t)\right)}_{Low}=\left\{ \begin{array}{ll} 1 & t\in [t_{3},t_{4}]\\ \frac{c_{ij}-\beta +\alpha *t}{c_{ij}-b_{ij}} & t\in [t_{2},t_{3}]\\ \;0 & \;other \end{array}\right.$$ (4)

$$\mu_{ij}{\left(x_{ij}(t)\right)}_{Medium}=\left\{ \begin{array}{ll} \frac{-\alpha *t+\beta -b_{ij}}{c_{ij}-b_{ij}} & t\in [t_{2},t_{3}]\\ \frac{d_{ij}-\beta +\alpha *t}{d_{ij}-c_{ij}} & t\in [t_{1},t_{2}]\\ 0 & \;other \end{array}\right.$$ (5)

$$\mu_{ij}{\left(x_{ij}(t)\right)}_{High}=\left\{ \begin{array}{ll} \frac{-\alpha *t+\beta -c_{ij}}{d_{ij}-c_{ij}} & t\in [t_{1},t_{2}]\\ 1 & t\in [t_{0},t_{1}]\\ 0 & \;other \end{array}\right.$$ (6)

By taking the first and second derivatives for Eqs (4)–(6), we have:

$$\frac{d\mu_{ij}{\left(x_{ij}(t)\right)}_{Low}}{dt}=\frac{\alpha }{c_{ij}-b_{ij}}>0\;and\;\frac{d^{\left(2\right)}\mu_{ij}{\left(x_{ij}(t)\right)}_{Low}}{dt^{2}}=0,\;t\in [t_{2},t_{3}]$$

$$\frac{d\mu_{ij}{\left(x_{ij}(t)\right)}_{Medium}}{dt}=\left\{ \begin{array}{cc} -\frac{\alpha }{c_{ij}-b_{ij}}<0, & t\in [t_{2},t_{3}]\\ \frac{\alpha }{d_{ij}-c_{ij}}>0, & t\in [t_{1},t_{2}] \end{array}\;and\;\frac{d^{\left(2\right)}\mu_{ij}{\left(x_{ij}(t)\right)}_{Medium}}{dt^{2}}=\{\begin{array}{c} 0,t\in [t_{2},t_{3}]\\ 0,t\in [t_{1},t_{2}] \end{array}\right.$$

$$\frac{d\mu_{ij}{\left(x_{ij}(t)\right)}_{High}}{dt}=-\frac{\alpha }{d_{ij}-c_{ij}}<0\;and\;\frac{d^{\left(2\right)}\mu_{ij}{\left(x_{ij}(t)\right)}_{High}}{dt^{2}}=0,t\in [t_{1},t_{2}]$$

Referring to bottom left of Fig 5, for the Low linguistic term *μ*_*ij*_(*x*_*ij*_(*t*))_*Low*_, it is increasing during the time interval [t_2_,t_3_] and approaches 1 in the interval [t_3_,t_4_]; as for the Medium linguistic term.

*μ*_*ij*_(*x*_*ij*_(*t*))_*Medium*_, it increases during the interval [t_1_,t_2_] and decreases on during the time interval [t_2_,t_3_]; for the High linguistic term *μ*_*ij*_(*x*_*ij*_(*t*))_*High*_, it decreases during the interval [t_1_,t_2_] and approaches 1 during the interval [t_0_,t_1_]. Apparently, as *x*_*ij*_(*t*) decreases over time, the Low and High linguistic terms exchange their relative location in the dynamic simulation environment, which consequently challenges the clustering of dynamic agents based on their dynamic membership values. Right side of Fig 5 provides a more complex case.

Example 2: Suppose the change of the linguistic variable *x*_*ij*_ over time is characterized by the function of *x*_*ij*_: g(t) = (upper right of Fig 4) where *α*,*β* > 0, the time horizon for the simulation is t_4_. We substitute *x*_*ij*_(*t*) with exp(β − α * t), now the corresponding membership functions over time are:

$$\mu_{ij}{\left(x_{ij}(t)\right)}_{Low}=\left\{ \begin{array}{ll} 1 & t\in [t_{3},t_{4}]\\ \frac{c_{ij}-e^{\left(\beta -\alpha *t\right)}}{c_{ij}-b_{ij}} & t\in [t_{2},t_{3}]\\ \;0 & \;other \end{array}\right.$$ (7)

$$\mu_{ij}{\left(x_{ij}(t)\right)}_{Medium}=\left\{ \begin{array}{ll} \frac{e^{\left(\beta -\alpha *t\right)}-b_{ij}}{c_{ij}-b_{ij}} & t\in [t_{2},t_{3}]\\ \frac{d_{ij}-e^{\left(\beta -\alpha *t\right)}}{d_{ij}-c_{ij}} & t\in [t_{1},t_{2}]\\ 0 & \;other \end{array}\right.$$ (8)

$$\mu_{ij}{\left(x_{ij}(t)\right)}_{High}=\left\{ \begin{array}{ll} \frac{e^{\left(\beta -\alpha *t\right)}-c_{ij}}{d_{ij}-c_{ij}} & t\in [t_{1},t_{2}]\\ 1 & t\in [t_{0},t_{1}]\\ \;0 & \;other \end{array}\right.$$ (9)

By taking the first and second derivatives of Eqs (7)–(9), we have:

$$\frac{d\mu_{ij}{\left(x_{ij}(t)\right)}_{Low}}{dt}=\frac{\alpha e^{\left(\beta -\alpha *t\right)}}{c_{ij}-b_{ij}}>0\;and\;\frac{d^{\left(2\right)}\mu_{ij}{\left(x_{ij}(t)\right)}_{Low}}{dt^{2}}=-\frac{\alpha^{2}}{c_{ij}-b_{ij}}e^{\left(\beta -\alpha *t\right)}<0,\;t\in [t_{2},t_{3}]$$

$$\frac{d\mu_{ij}{\left(x_{ij}(t)\right)}_{Medium}}{dt}\left\{ \begin{array}{ll} \frac{{-\alpha e}^{\left(\beta -\alpha *t\right)}}{c_{ij}-b_{ij}}<0, & t\in [t_{2},t_{3}]\\ \frac{\alpha e^{\left(\beta -\alpha *t\right)}}{d_{ij}-c_{ij}}>0, & t\in [t_{1},t_{2}] \end{array}\right.\;and$$

$$\frac{d^{\left(2\right)}\mu_{ij}{\left(x_{ij}(t)\right)}_{Medium}}{dt^{2}}=\left\{ \begin{array}{l} \frac{\alpha^{2}}{c_{ij}-b_{ij}}e^{\left(\beta -\alpha *t\right)}>0,\;t\in [t_{2},t_{3}]\\ -\frac{\alpha^{2}}{d_{ij}-c_{ij}}e^{\left(\beta -\alpha *t\right)}<0,t\in [t_{1},t_{2}] \end{array}\right.$$

$$\frac{d\mu_{ij}{\left(x_{ij}(t)\right)}_{High}}{dt}=-\frac{{\alpha e}^{\left(\beta -\alpha *t\right)}}{d_{ij}-c_{ij}}<0\;and\;\frac{d^{\left(2\right)}\mu_{ij}{\left(x_{ij}(t)\right)}_{High}}{dt^{2}}=\frac{\alpha^{2}}{d_{ij}-c_{ij}}e^{\left(\beta -\alpha *t\right)}>0,t\in [t_{1},t_{2}]$$

The linguistic term *μ*_*ij*_(*x*_*ij*_(*t*))_*Low*_ increases and is concave downward in the interval [t_2_,t_3_] and approaches 1 in the interval [t_3_,t_4_]; *μ*_*ij*_(*x*_*ij*_(*t*))_*Medium*_ decreases and is concave upwards during the interval [t_2_,t_3_] and increases and is concave downwards in the interval [t_1_,t_2_]; *μ*_*ij*_(*x*_*ij*_(*t*))_*High*_ decreases and is concave upwards in the interval [t_1_,t_2_] and approaches 1 in the interval [t_0_,t_1_]. In the system dynamics model, the behavior of the state variables can be more complicated than what we have shown in examples 1 and 2.

#### Peer effects on interacting agents—second dynamic behavior

In the proposed hybrid model, there are generally two types of interactions among agents. One case is that agents randomly make contact with certain (e.g., three persons) agents per day and impact each other by exchanging information and changing their preference accordingly. The other case is that agents have a preset relationship with certain agents in a scale-free network [48,49], which means that in the community or designated population a few agents are at the hub of the network and the rest agents do not have many connections. In a scale-free network, the degree distribution follows a power law, i.e. P(k)∼k − q, where k is degree (1 < *k* < ∞) and *q* is a parameter with value in the range (2 < *q* < 3).

Although we demonstrate distinct interacting mechanisms among agents for the abovementioned two cases, the operations associated with multiple linguistic variables characterizing agents in general do not have much difference. For handling the interactions of fuzzy agents, we need to define the rules, i.e., the way in which agents interact and the results that will be obtained as a result of the interactions.

Rule 1: Choosing linguistic term to start with. During the intervention implementation process, in order to achieve the desired outcomes under scarce resources, it is necessary for decision makers to allocate resource to those being worse off as a consequence of previous policies or decisions. For example, minimizing *μ*_*ij*_(*x*_*ij*_(*t*))_*Low*_ (e.g., low satisfaction with respect to the outdoor activity space) with high degree of membership could be the priority. Therefore, we simplify the operations of multiple linguistic variables by only taking into consideration the operations of the Low linguistic term of each linguistic variable, which can be easily extended to include other linguistic terms if necessary.

Rule 2: Fuzzy inference. It is assumed that on the average agent makes contact with other k agents per time unit (generally *k* ≤ 5, time unit = unit of time in the simulation) in a random manner. And it is assumed that the model is simulated over n time units and the start time is defined *t*_0_ and end time is *p* * *τ*. Three inference rules are defined hereby.

Pessimistic case. In this case, the obtained information from the peer agent negatively affects an agent's decision. Having communicated with other agents, the agent will choose to attain the lower value among them, i.e., the updated value of *μ*_*ij*_(*x*_*ij*_(*t*_0_))_*Low*_ should take the smaller value obtained by them when comparing itself *i*^*th*^ agent with the interacting agents (*i* − *r*)^*th*^ agent (we assume contact for 5 times).

$$\mu_{ij}{\left(x_{ij}(t_{0})\right)}_{Low}\to \mu_{ij}{\left(x_{ij}(t_{1})\right)}_{Low}=min\{\mu_{ij}{\left(x_{ij}(t_{1})\right)}_{Low},\mu_{(i-r)j}{\left(x_{(i-r)j}(t_{1})\right)}_{Low}\}$$

$$\mu_{(i-r)j}{\left(x_{(i-r)j}(t_{0})\right)}_{Low}{\to \mu }_{(i-r)j}{\left(x_{(i-r)j}(t_{1})\right)}_{Low}=min\{\mu_{ij}{\left(x_{ij}(t_{1})\right)}_{Low},\mu_{(i-r)j}{\left(x_{(i-r)j}(t_{1})\right)}_{Low}\}$$

**Fig 6:**
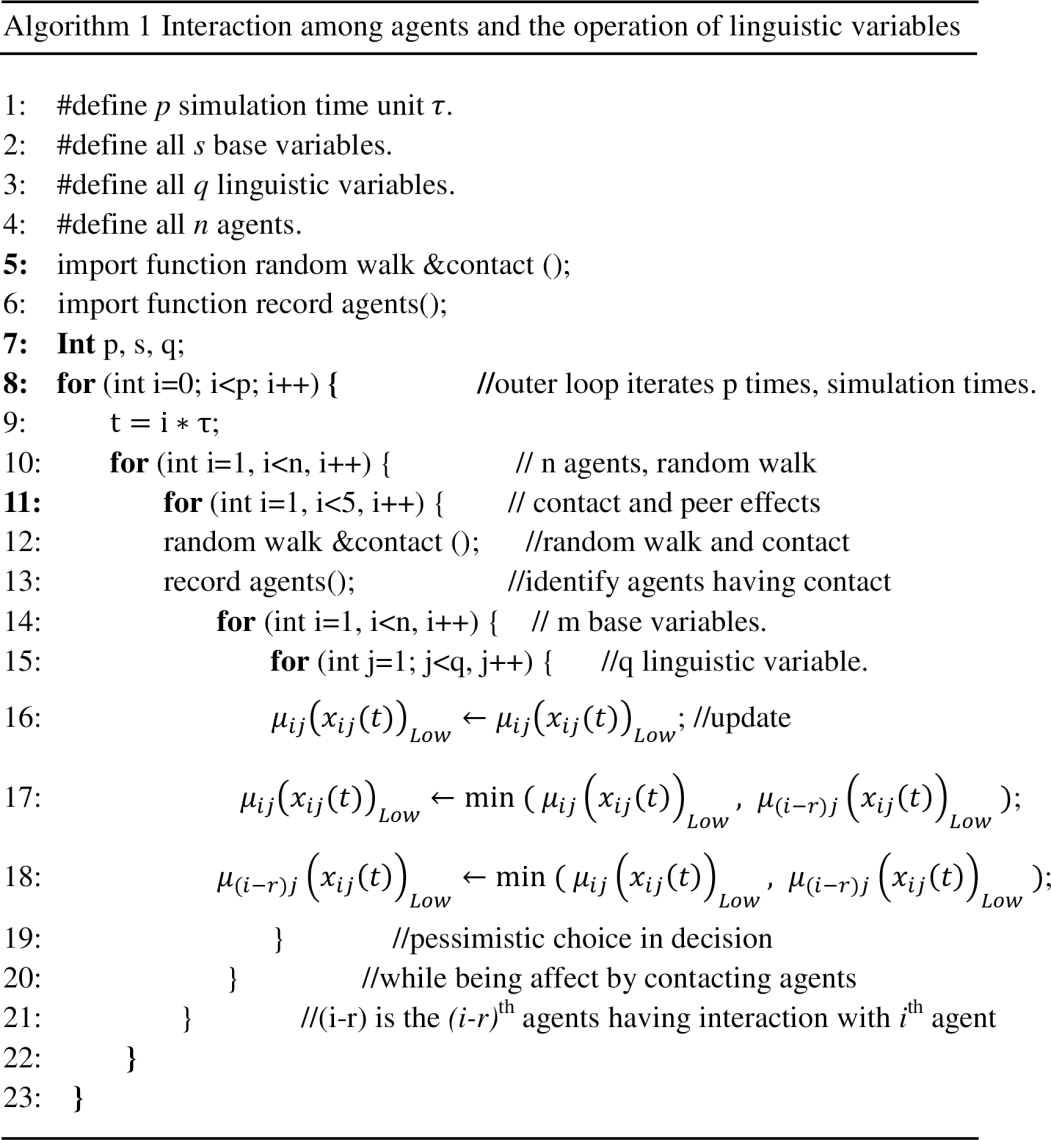
Pseudo code for the interactions among agents and the operations of linguistic variables.

Please refer to Fig 6 for Algorithm 1.

Optimistic case. In this case, the updated value of *μ*_*ij*_(*x*_*ij*_(*t*_0_))_*Low*_ will take the higher value obtained by them when comparing itself *i*^*th*^ agent to the interacting agents (*i* − *r*)^*th*^ agent (we assume contact for 5 times).

$$\mu_{ij}{\left(x_{ij}(t_{0})\right)}_{Low}\to \mu_{ij}{\left(x_{ij}(t_{1})\right)}_{Low}=max\{\mu_{ij}{\left(x_{ij}(t_{1})\right)}_{Low},\mu_{(i-r)j}{\left(x_{(i-r)j}(t_{1})\right)}_{Low}\}$$

$$\mu_{(i-r)j}{\left(x_{(i-r)j}(t_{0})\right)}_{Low}\to \mu_{(i-r)j}{\left(x_{(i-r)j}(t_{1})\right)}_{Low}=max\{\mu_{ij}{\left(x_{ij}(t_{1})\right)}_{Low},\mu_{(i-r)j}{\left(x_{(i-r)j}(t_{1})\right)}_{Low}\}$$

Realistic (mediocre) case. In this case, the updated value of *μ*_*ij*_(*x*_*ij*_(*t*_0_))_*Low*_ will take the weighted sum of both values obtained by them. The assignment of weights *φ*, ω is determined by the constants that define the triangular membership functions for the linguistic variables (Fig 3), which provides the boundary for different the linguistic terms. Based on the definitions, the larger the constants are the less sensitive the agent responds to the change of the state variables (interventions). Upon an interaction, the agent tends to reduce the gap between itself and a peer [50]. Therefore, in the realistic case, an agent with smaller constants that define the linguistic variables attempts to assign larger values to the interacting agent with larger constants for the linguistic variables, i.e. *φ*_i_ < ω_*i*_ if b_(i−r)j_ > b_ij_.

$$\mu_{ij}{\left(x_{ij}(t_{0})\right)}_{Low}\to \mu_{ij}{\left(x_{ij}(t_{1})\right)}_{Low}=\varphi_{i}\mu_{ij}{\left(x_{ij}(t_{1})\right)}_{Low}+{\omega_{i}\mu }_{(i-r)j}{\left(x_{(i-r)j}(t_{1})\right)}_{Low}$$

$$\mu_{(i-r)j}{\left(x_{(i-r)j}(t_{0})\right)}_{Low}{\to \mu }_{(i-r)j}{\left(x_{(i-r)j}(t_{1})\right)}_{Low}={\varphi_{i-r}\mu }_{ij}{\left(x_{ij}(t_{1})\right)}_{Low}+{\omega_{i-r}\mu }_{(i-r)j}{\left(x_{(i-r)j}(t_{1})\right)}_{Low}$$

#### Clustering of fuzzy agents in subspaces

Having completed the interactions and information exchange, agents will be clustered in different subspaces based on the linguistic variables that characterize them, which is conducted in a virtual manner (shadow variable) since it is not possible to physically separate an agent into several parts. That is to say, agents could be virtually located in several subspaces when the clustering operation is completed (Fig 5).

In the previous discussion, we simplified the use of linguistic variables to the linguistic term "Low" to reflect how an agent responds to interventions, where *μ*_*ij*_(*x*_*ij* (*p* * *τ*))_*Low* ∈ [0,1] is the final value (after simulation) for the j^th^ linguistic variable of i^th^ agent. It is assumed in following discussion that there are 2 base variables, i.e., two state variables *sv*_*l*_(*t*) and *sv*_*m*_(*t*) that reflect the intervention results in the SD model. Notwithstanding the simplification, this research still faces the challenge of dealing with operations and the clustering of complex high-dimensional data.

**Fig 7:**
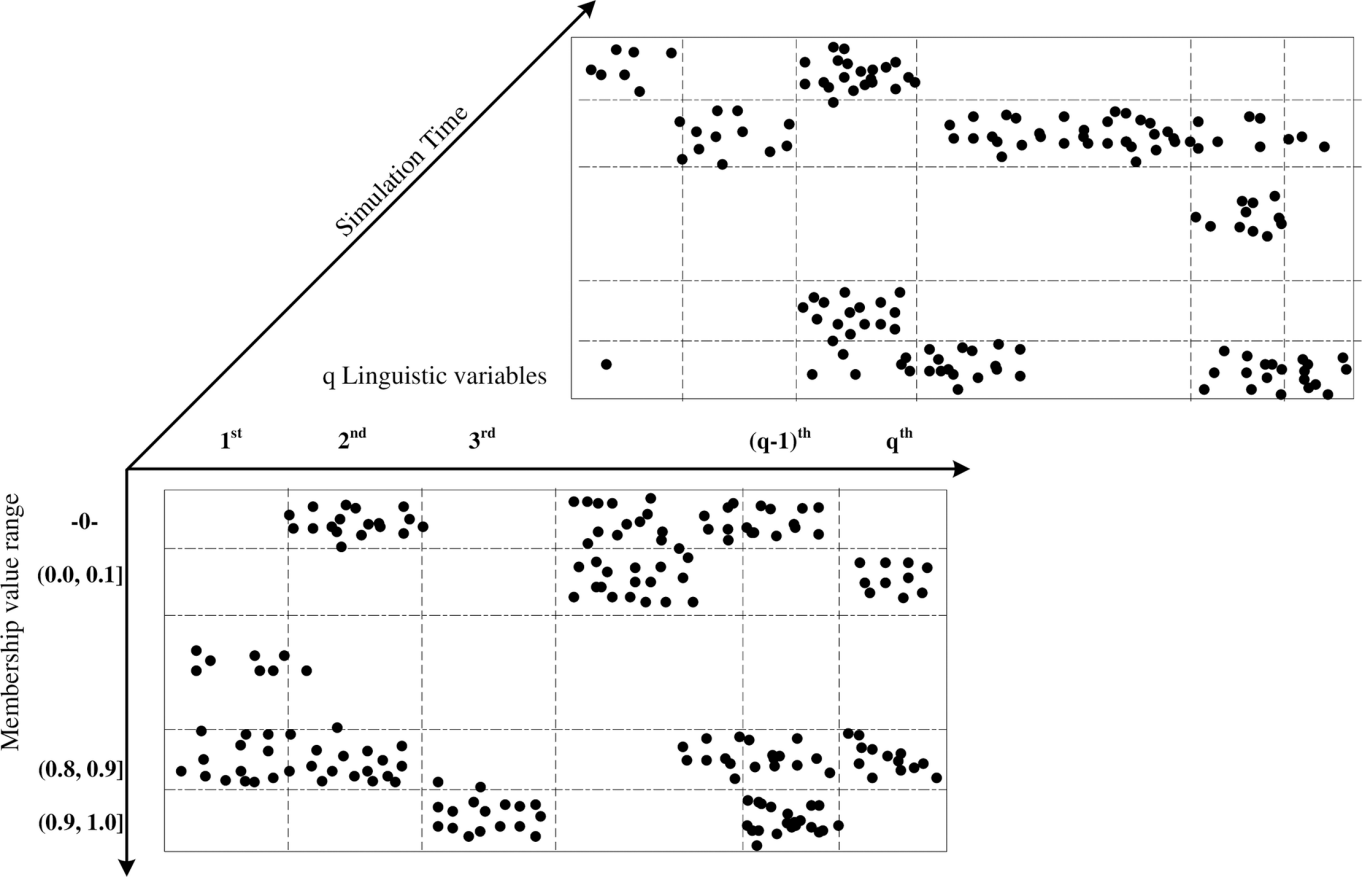
Clustering of fuzzy agents in subspaces.

Given the fact that the input of resources corresponds the membership value that μ_ij_(*x*_*ij* (*p* * *τ*))_*Low* actually takes, we use the following intervals to cluster these agents, i.e. 0, (0,0.1], (0.1,0.2],…, (0.8,0.9], (0.9,1.0] (Fig 7).

**Fig 8:**
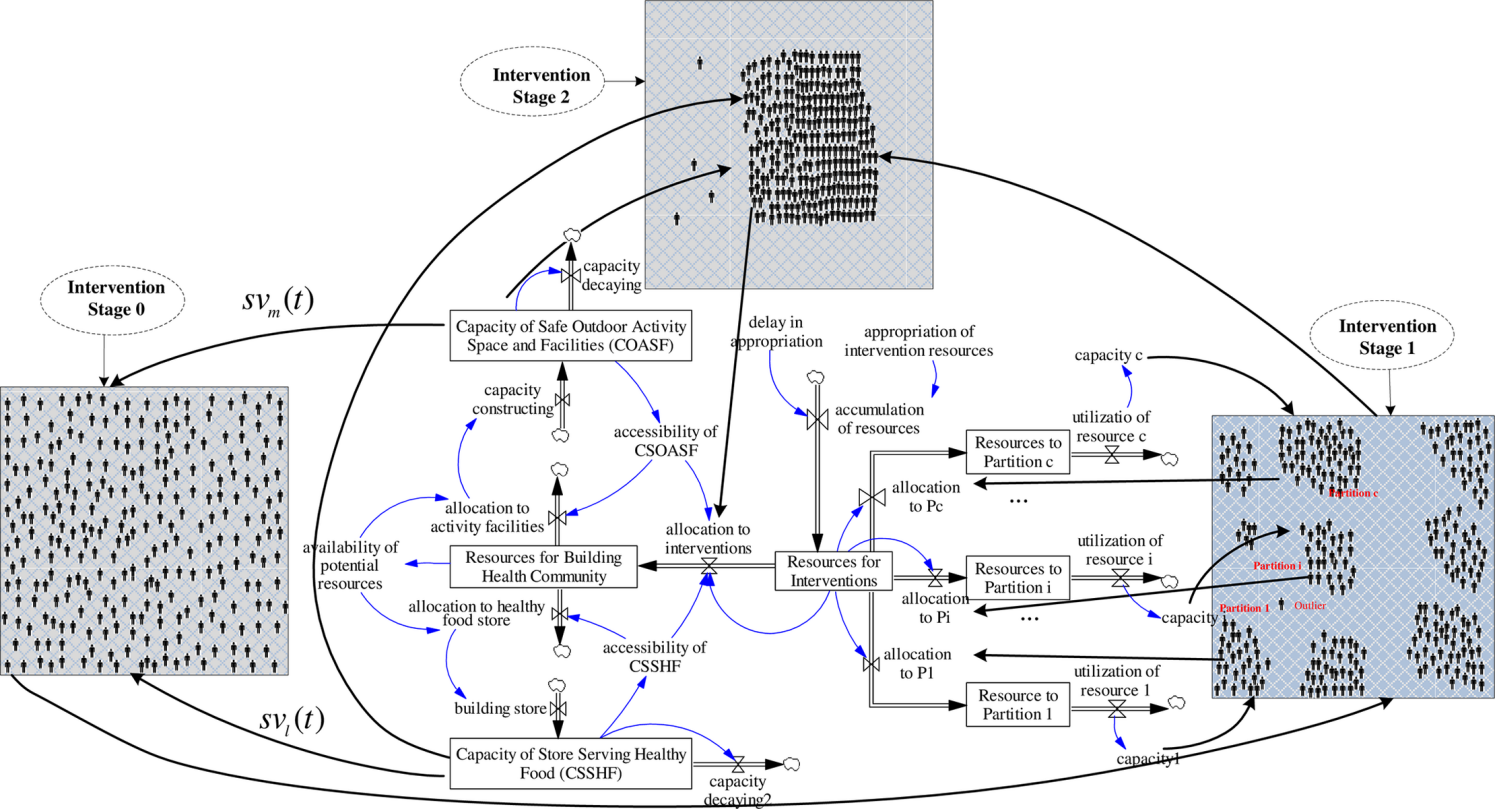
Hybrid simulation model by combining agent-based simulation and system dynamic modeling.

For agents having linguistic variables falling into the membership range 0, there is no need to allocate resources to these agents. For those who fall into other ranges, their corresponding discrepancies will be fed back to the system dynamics model for next stage simulation, i.e., from Stage0-Stage1 (Fig 8). With a few iterations, most of the agents will become homogeneous (i.e. Stage2 status in Fig 8).

### Institutional resource allocation decision-making

#### Incorporating linguistic variable into system dynamics model

**Fig 9:**
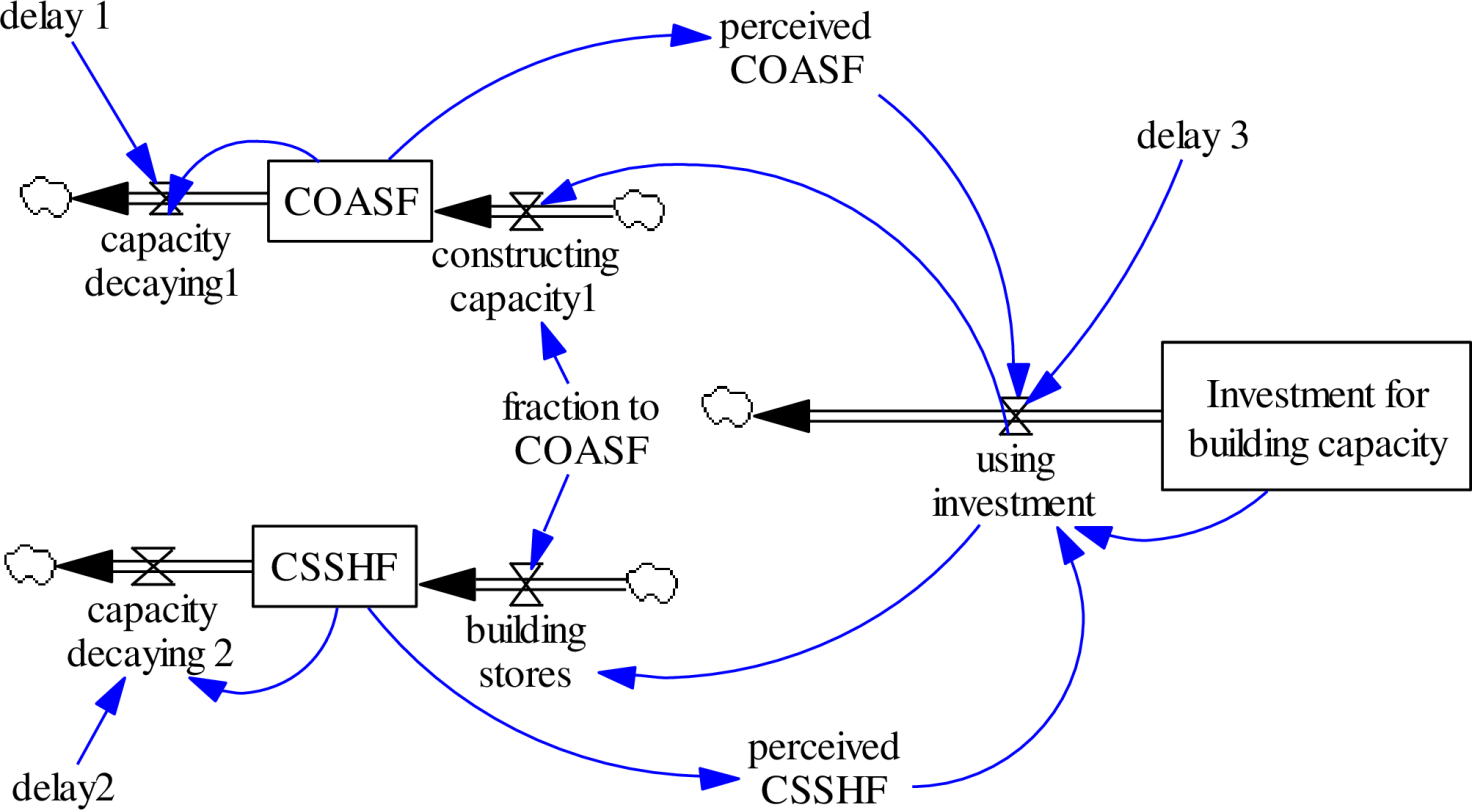
Simplified system dynamics model with two linguistic variables.

The SD model part of the proposed hybrid model is responsible for the resource allocations. In this part, two linguistic variables with respect two different base variables are investigated. This SD model is built on two previous mature models [40, 51]. We also borrowed a partial dataset from them since there is no such implementation for a real case. Fig 9 provides a simplified version that demonstrates how to incorporate linguistic variables into a system dynamics model.

In Fig 9, two state variables COASF − *s*_1_(*t*) and CSSHF − *s*_2_(*t*) are increased by the inflow of building the capacity (*φ*_1_ and *φ*_2_) and are decreased by the outflow of capacity decaying (*λ*_1_ and *λ*_2_) where the COASF is the “Capacity of Safe Outdoor Activity Space and Facilities” and CSSHF is the “Capacity of Store Serving Healthy Food”. Intervention implementers make decision based on the interactions of the perceived values of two state variables, which decide how much money will be used for building the capacity of stock (state variables) with fraction *p* and 1 − *p*. Based on their expertise and past experiences, they will define a set of rules when manipulating two linguistic variables while considering their significance.

$$s_{1}(t)=\int_{0}^{t}{(\varphi }_{1-}\lambda_{1})dt$$ (10)

$$s_{2}(t)=\int_{0}^{t}{(\varphi }_{2-}\lambda_{2})dt$$ (11)

where $\lambda_{1}=\frac{s_{1}(t)}{d_{1}}$, $\lambda_{2}=\frac{s_{2}(t)}{d_{2}}$, *d*_1_ and *d*_2_ are life years of built capacities.

The inflows *φ*_1_ and *φ*_2_ are determined by the allocation fraction *p* and 1 − *p* and flow of "using investment" ω(*t*).

$$\varphi_{1}=\omega (t)\times p$$ (12)

$$\varphi_{2}=\omega (t)\times (1-p)$$ (13)

Flow ω(*t*) is determined by perceived values of *x* and *y*, delay3 (*d*_3_), and "Investment for building capacity *ρ*(*t*)". It is assumed that perceived values of *x*, *y* are defined by triangular membership functions as follows

$$\mu {\left(x\right)}_{Low}=\left\{ \begin{array}{ll} \;1 & if\;x\in [0,b_{i}],b_{i}>0\\ \frac{c_{i}-x}{c_{i}-b_{i}} & if\;x\in [b_{i},c_{i}],0<b_{i}<c_{i}\\ \;0 & \;other \end{array}\right.$$ (14)

$$\mu {\left(x\right)}_{Medium}=\left\{ \begin{array}{ll} \frac{x-b_{i}}{c_{i}-b_{i}} & if\;x\in [b_{i},c_{i}],0<b_{i}<c_{i}\\ \frac{d-x}{d_{i}-c_{i}} & if\;x\in [c_{i},d_{i}],0<c_{i}<d_{i}\\ \;0 & \;other \end{array}\right.$$ (15)

$$\mu {\left(x\right)}_{High}=\left\{ \begin{array}{ll} \frac{x-c_{i}}{d_{i}-c_{i}} & if\;x\in [c_{i},d_{i}],0<c_{i}<d_{i}\\ \;1 & \;if\;x\in [d_{i},+\infty ]\\ 0 & \;other \end{array}\right.$$ (16)

In order to incorporate the two linguistic variables, we need to define rules for the operations of two fuzzy variables (refer to Table 1).

**Table 1:** Fuzzy rule definition.

Rule no.	Perceived COASF	Perceived CSSHF	Perceived impact on investment
**Rule1**	Low	Low	Low
**Rule2**	Low	Medium	Low
**Rule3**	Low	High	Medium
**Rule4**	Medium	Low	Low
**Rule5**	Medium	Medium	Medium
**Rule6**	Medium	High	Medium
**Rule7**	High	Low	High
**Rule8**	High	Medium	High
**Rule9**	High	High	High

In the system dynamics model, we defined two linguistic variables COASF and CSSHF, each having three linguistic terms. Therefore, in order to fully consider their mutual interaction, nine rules (3 × 3) are evaluated in our model. In the fuzzy logic system, fuzzy rules defined generally represent the knowledge, experiences, and expertise of decision makers or policy makers. Those rules are derived through several mechanisms 1) verbalizing decision maker’s experiences and expertise, 2) conducting a dedicated survey, 3) observing decision makers' behaviors, 4) tracing behavior with wearable sensors, and 5) textual analysis. As decision makers consciously or unconsciously use such rules to respond to particular interventions and/or actions, rules can be recorded when observing their behaviors [29, 40, 52, 53, 54].

**Fig 10:**
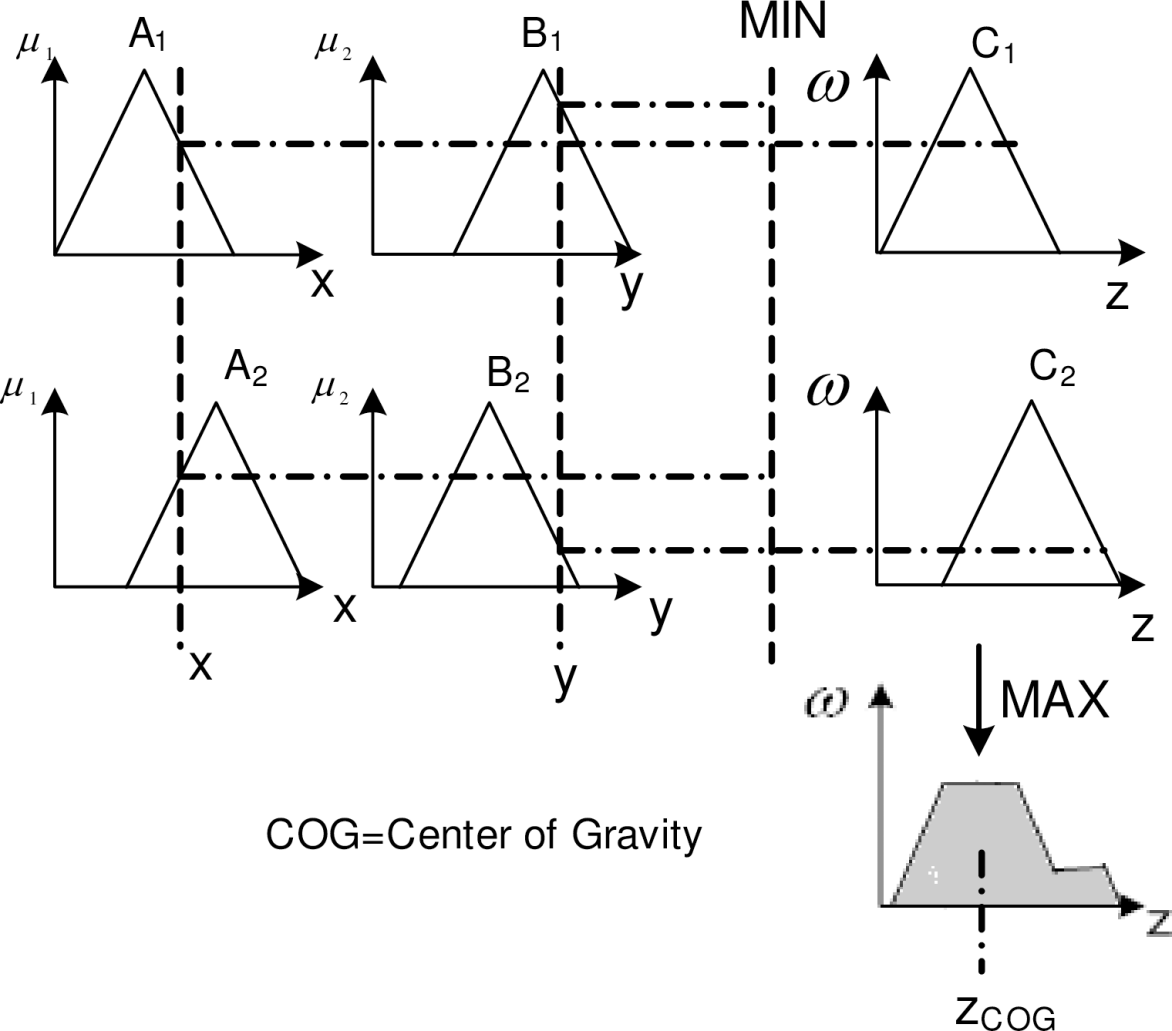
Consequence of the rule evaluation of the decision-making process.

The mechanism of the Mamdani controller [55] is used to get the results based on rule evaluations. For instance, two rules apply and are evaluated at certain time *τ* (referring to Fig 10). By using the center of gravity (COG), we obtain the value *z*_*COG*_ (*z* represents the rule consequence of fuzzy variables).

$$z_{COG}=\frac{\int_{a}^{b}z\mu (z)dz}{\int_{a}^{b}\mu (z)dz}$$ (17)

$$\rho (t)=C-\int_{0}^{t}\omega (t)dt$$ (18)

where C is the initial fund in the investment stock,

$$\omega (t)=z_{COG}\times \frac{\rho (t)}{d_{3}}$$ (19)

Substitute (18) into (19) to solve for *ω*(*t*),

$$\omega (t)=e^{\left(t+\frac{z_{COG}}{d_{3}}\right)}$$ (20)

$$\varphi_{1}=e^{\left(t+\frac{z_{COG}}{d_{3}}\right)}\times p$$ (21)

$$\varphi_{2}=e^{\left(t+\frac{z_{COG}}{d_{3}}\right)}\times (1-p)$$ (22)

$$s_{1}(t)=\int_{0}^{t}{(e^{\left(t+\frac{COG}{d_{3}}\right)}\times p}_{-}\frac{s_{1}(t)}{d_{1}})dt$$ (23)

$$s_{2}(t)=\int_{0}^{t}(e^{\left(t+\frac{z_{COG}}{d_{3}}\right)}\times (1-p)-\frac{s_{2}(t)}{d_{2}})dt$$ (24)

Take the Laplace transform for (23) and (24), we have

$$s_{1}(t)=d_{1}(1-\frac{1}{e^{d_{1}}})\times \int_{0}^{t}{(e}^{\left(t+\frac{COG}{d_{3}}\right)}\times p)dt$$ (25)

$$s_{2}(t)=d_{2}(1-\frac{1}{e^{d_{2}}})\times \int_{0}^{t}{(e}^{\left(t+\frac{COG}{d_{3}}\right)}\times (p-1))dt$$ (26)

## Results

**Fig 11:**
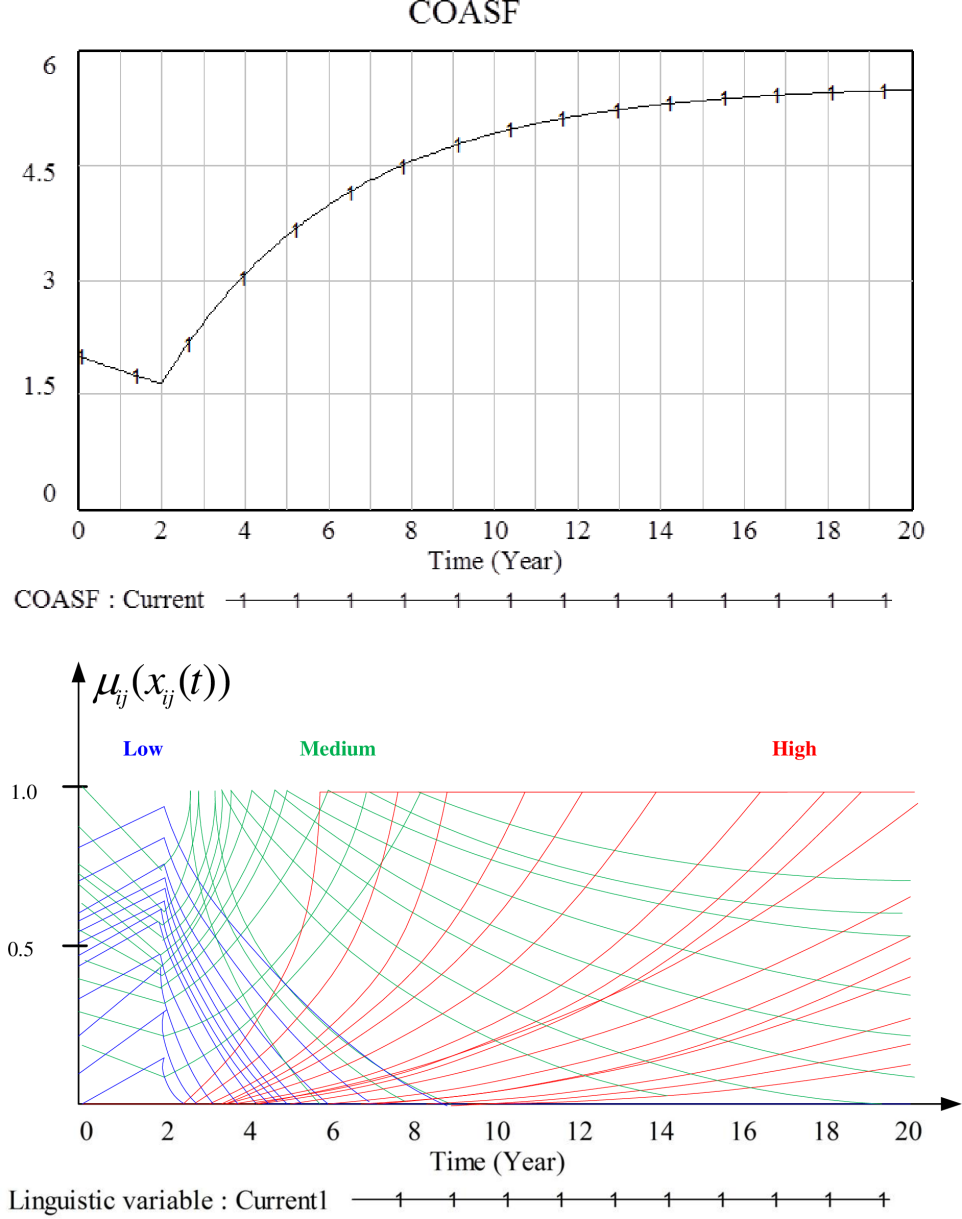
Behaviors of capacity of safe outdoor activity space and facilities (COASF) in the system dynamics model and corresponding values of the linguistic variables in the agent-based model.

**Fig 12:**
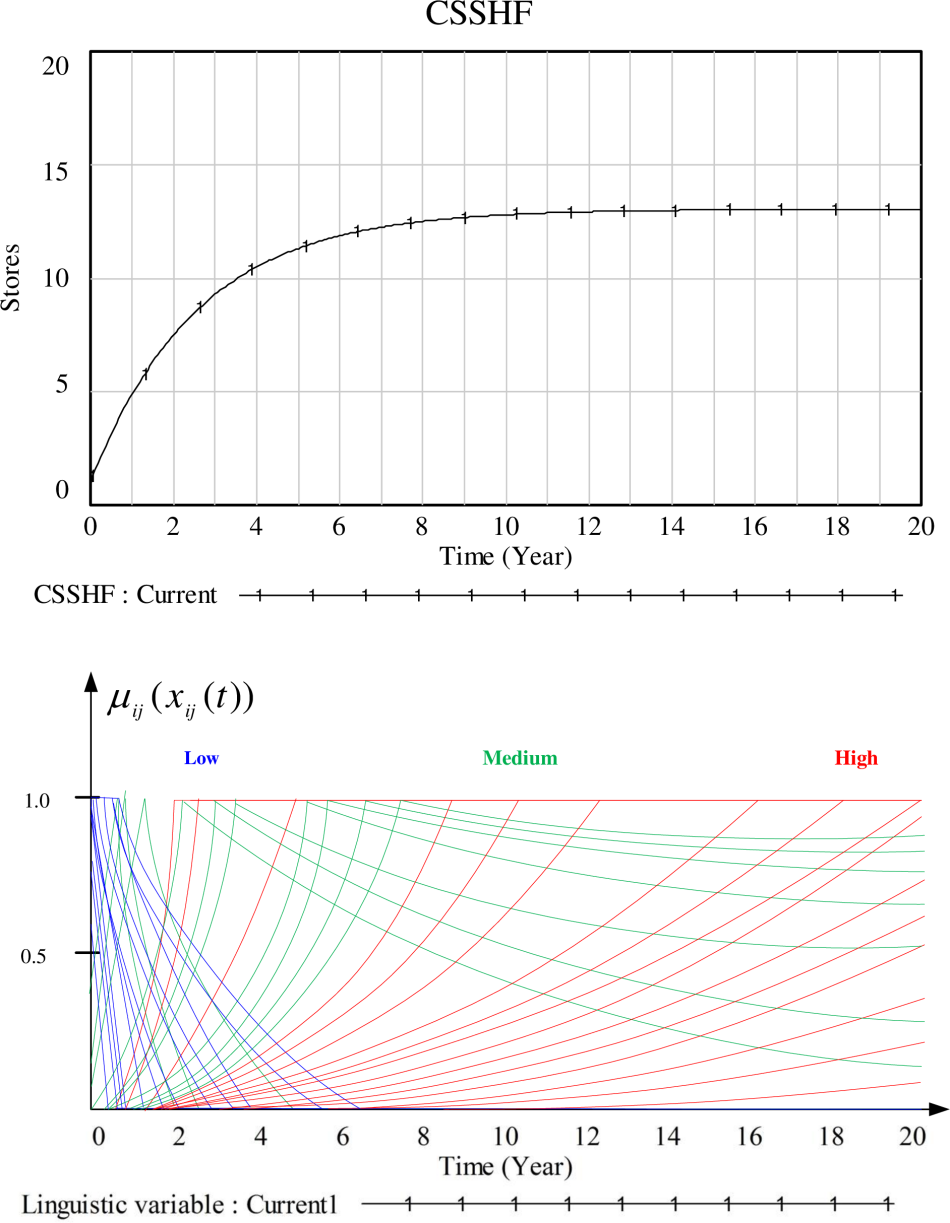
Behaviors of capacity of store serving healthy food (CSSHF) in the system dynamics model and corresponding values of the linguistic variables in agent-based model.

Having discussed all mathematical formulations involved in this hybrid model, Figs 11 and 12 demonstrate the behaviors of two linguistic variables, i.e. COASF and CSSHF (state variables) and partial values of the different terms of the linguistic variables. As mentioned in the previous section, the constants defining the linguistic variables of different agents follow the normal distribution. This is why the obtained values of these linguistic variables behave as represented in the Figs 11 and 12, i.e., multiple lines representing different linguistic terms of different agents. As the display of a whole set will make the line unidentifiable, therefore we just show some typical curves. Based on the aforementioned clustering criteria, these agents could be grouped into different clusters preparing for further interventions.

## Discussion

Due to the unavailability of commercial off-the-shelf simulation tools that can incorporate fuzzy logic into a hybrid model of SDM and ABM, only partial results were demonstrated, which makes the objective of capturing multistage fuzzy uncertainties in decision making for both individuals and institutions very challenging. Although Anylogic^®^ simulation platform does provide limited capability of modeling a hybrid ABM/SD model that incorporates fuzzy logic into the hybrid model, it is not capable of capturing and depicting very complicated dynamics taking into account the uncertainties represented by the linguistic variables for both the agent decision making process and the decision making associated with institutions. It is even harder for the software to carry out optimizations (e.g., resource allocation decisions) in the hybrid model that takes into account multistage uncertainties.

With respect to the modeling of the intervention path (refer to Fig 8), this framework has assumed the path starting from implementing interventions on heterogeneous individuals. These individuals then form different clusters due to the impacts from the interventions and interaction effects (peer effects and communications). Having allocated the necessary resources for the designated clusters for precise intervention (i.e., resource allocation is not evenly distributed to clusters with different characteristics but based on the actual need of a particular group), distinct clusters converge to a single homogeneous cluster for a set of characteristics for which the policy makers or implementers are concerned with. A second intervention path could start by implementing interventions on population with homogeneous characteristics and then focus on individuals with heterogeneous interests. With the ensuing interventions, the impacts on individuals, and influence of agent interactions, the agents form clusters. Additional resource allocations render the clusters with distinct features homogeneous. The last but not the least intervention path could start by implementing interventions on multiple clusters. In this scenario, two dynamics could be investigated with one focusing on modeling the impacts of precisely targeted interventions on individuals of a particular cluster and the other focusing on modeling the dynamic interactions of heterogeneous clusters.

With respect to optimization in the hybrid model, multiple objectives could be considered. The first consideration is to maximize the implementation effectiveness given certain amount of resources appropriated for the intervention. The second consideration is to minimize resources used for interventions by accurately pinpointing pertinent (i.e., satisfying amount of resources for particular group of agents with certain linguistic characteristics) clusters and using appropriate resources accordingly. The third consideration is to maximize implementation effectiveness by optimizing resource allocation for the combination of multiple interventions by identifying characteristics of clusters and implementing precise interventions accordingly.

The next challenge in using this fuzzy hybrid model is to consider the complexity of obtaining big data and converting big data, especially unstructured data into formats that can be used in the simulation model. Since Big data-driven research brings important opportunities for the precise intervention and for customized individual-based healthcare [56–58], it helps inform implementation research. The biggest challenge lies that how to translate (textual and linguistic variables) and integrate multiple streams of live population data into simulation environment and generate meaning outputs for support implementation of policies and interventions. In order to capture the uncertainties represented by fuzzy variables at different stages for both the individual and institutional decision making, it is necessary to identify pertinent fuzzy variables and corresponding linguistic values. The questions that need to be answered include but not limited to: which fuzzy variables are critical for tracking the effectiveness of the intervention impact and their use of resources; what are the appropriate dimensions of a fuzzy variable, i.e., what are the appropriate states of human perception with respect to a decision variable (e.g., satisfaction with respect to treatment); how should interacting rules (agent interaction, cluster interaction) be defined when considering multiple fuzzy variables with multiple linguistic values; how should optimization be explored considering the existence of many states of human perception with respect to decision variables and their complex combinations?

Even though many challenges exist, it is very promising to use a fuzzy hybrid model to simultaneously capture heterogeneity and homogeneity and fuzzy uncertainty in the implementation of interventions in complex socioeconomic systems such as transportation system.

## Conclusions

This research investigates the use of linguistic variables in multiple stages of a hybrid model that combines an agent-based simulation model and a system dynamics model. We systematically present how to handle high-dimensional unstructured data associated with the implementation of public interventions. This research provides a mathematical framework when modeling and simulating complex socioeconomic systems, and capturing uncertainties using fuzzy logic. This research raises some challenges for in-depth research to consider system optimization, complex interacting mechanisms (networks), increased agent attributes, and the tradeoff between the validity of the results and the requisite modeling efforts.

## Supporting information

S1 Dataset Dataset used for generating Figs 11 and 12. (DOCX)

## Abbreviations

COASF: Capacity of Safe Outdoor Activity Space and Facilities
CSSHF: Capacity of Store Serving Healthy Food
