# Supplementary material for: Capturing multi-stage fuzzy uncertainties in hybrid system dynamics and agent-based models for enhancing policy implementation in health systems research
Source: PLoS One. 2018 Apr 25;13(4):e0194687. doi: 10.1371/journal.pone.0194687 (PMC5918643; doi:10.1371/journal.pone.0194687)
Supplement: S1 Dataset — (DOCX) [file pone.0194687.s001.docx]

Table A1. Dataset for generating Fig 11

| **Time (Year)** | **COASF** | **µ_ij_(x_ij_(t))_Low_** | **µ_ij_(x_ij_(t))_M_** | **µ_ij_(x_ij_(t))_H_** |
| --- | --- | --- | --- | --- |
| 0 | 2 | 0.2 | 0.8 |  |
| 0.25 | 1.95 | 0.22 | 0.78 |  |
| 0.5 | 1.90125 | 0.2395 | 0.7605 |  |
| 0.75 | 1.85372 | 0.258512 | 0.741488 |  |
| 1 | 1.80738 | 0.277048 | 0.722952 |  |
| 1.25 | 1.76219 | 0.295124 | 0.704876 |  |
| 1.5 | 1.71814 | 0.312744 | 0.687256 |  |
| 1.75 | 1.67518 | 0.329928 | 0.670072 |  |
| 2 | 1.6333 | 0.34668 | 0.65332 |  |
| 2.25 | 1.85393 | 0.258428 | 0.741572 |  |
| 2.5 | 2.06215 | 0.17514 | 0.82486 |  |
| 2.75 | 2.25865 | 0.09654 | 0.90346 |  |
| 3 | 2.4441 | 0.02236 | 0.97764 |  |
| 3.25 | 2.61912 | 0 | 0.952352 | 0.047648 |
| 3.5 | 2.7843 | 0 | 0.88628 | 0.11372 |
| 3.75 | 2.94018 | 0 | 0.823928 | 0.176072 |
| 4 | 3.08729 | 0 | 0.765084 | 0.234916 |
| 4.25 | 3.22613 | 0 | 0.709548 | 0.290452 |
| 4.5 | 3.35716 | 0 | 0.657136 | 0.342864 |
| 4.75 | 3.48082 | 0 | 0.607672 | 0.392328 |
| 5 | 3.59753 | 0 | 0.560988 | 0.439012 |
| 5.25 | 3.70767 | 0 | 0.516932 | 0.483068 |
| 5.5 | 3.81161 | 0 | 0.475356 | 0.524644 |
| 5.75 | 3.90971 | 0 | 0.436116 | 0.563884 |
| 6 | 4.00229 | 0 | 0.399084 | 0.600916 |
| 6.25 | 4.08966 | 0 | 0.364136 | 0.635864 |
| 6.5 | 4.17211 | 0 | 0.331156 | 0.668844 |
| 6.75 | 4.24993 | 0 | 0.300028 | 0.699972 |
| 7 | 4.32337 | 0 | 0.270652 | 0.729348 |
| 7.25 | 4.39268 | 0 | 0.242928 | 0.757072 |
| 7.5 | 4.4581 | 0 | 0.21676 | 0.78324 |
| 7.75 | 4.51983 | 0 | 0.192068 | 0.807932 |
| 8 | 4.57809 | 0 | 0.168764 | 0.831236 |
| 8.25 | 4.63307 | 0 | 0.146772 | 0.853228 |
| 8.5 | 4.68496 | 0 | 0.126016 | 0.873984 |
| 8.75 | 4.73393 | 0 | 0.106428 | 0.893572 |
| 9 | 4.78015 | 0 | 0.08794 | 0.91206 |
| 9.25 | 4.82376 | 0 | 0.070496 | 0.929504 |
| 9.5 | 4.86493 | 0 | 0.054028 | 0.945972 |
| 9.75 | 4.90378 | 0 | 0.038488 | 0.961512 |
| 10 | 4.94044 | 0 | 0.023824 | 0.976176 |
| 10.25 | 4.97504 | 0 | 0.009984 | 0.990016 |
| 10.5 | 5.00769 | 0 | 0 | 1 |
| 10.75 | 5.03851 | 0 | 0 | 1 |
| 11 | 5.06759 | 0 | 0 | 1 |
| 11.25 | 5.09504 | 0 | 0 | 1 |
| 11.5 | 5.12095 | 0 | 0 | 1 |
| 11.75 | 5.14539 | 0 | 0 | 1 |
| 12 | 5.16846 | 0 | 0 | 1 |
| 12.25 | 5.19024 | 0 | 0 | 1 |
| 12.5 | 5.21079 | 0 | 0 | 1 |
| 12.75 | 5.23018 | 0 | 0 | 1 |
| 13 | 5.24848 | 0 | 0 | 1 |
| 13.25 | 5.26576 | 0 | 0 | 1 |
| 13.5 | 5.28206 | 0 | 0 | 1 |
| 13.75 | 5.29744 | 0 | 0 | 1 |
| 14 | 5.31196 | 0 | 0 | 1 |
| 14.25 | 5.32566 | 0 | 0 | 1 |
| 14.5 | 5.33859 | 0 | 0 | 1 |
| 14.75 | 5.3508 | 0 | 0 | 1 |
| 15 | 5.36232 | 0 | 0 | 1 |
| 15.25 | 5.37319 | 0 | 0 | 1 |
| 15.5 | 5.38344 | 0 | 0 | 1 |
| 15.75 | 5.39312 | 0 | 0 | 1 |
| 16 | 5.40226 | 0 | 0 | 1 |
| 16.25 | 5.41088 | 0 | 0 | 1 |
| 16.5 | 5.41902 | 0 | 0 | 1 |
| 16.75 | 5.4267 | 0 | 0 | 1 |
| 17 | 5.43395 | 0 | 0 | 1 |
| 17.25 | 5.44079 | 0 | 0 | 1 |
| 17.5 | 5.44725 | 0 | 0 | 1 |
| 17.75 | 5.45334 | 0 | 0 | 1 |
| 18 | 5.45909 | 0 | 0 | 1 |
| 18.25 | 5.46451 | 0 | 0 | 1 |
| 18.5 | 5.46964 | 0 | 0 | 1 |
| 18.75 | 5.47447 | 0 | 0 | 1 |
| 19 | 5.47903 | 0 | 0 | 1 |
| 19.25 | 5.48333 | 0 | 0 | 1 |
| 19.5 | 5.4874 | 0 | 0 | 1 |
| 19.75 | 5.49123 | 0 | 0 | 1 |
| 20 | 5.49485 | 0 | 0 | 1 |

Table A2. Dataset for generating Fig 12

| **Time (Year)** | **CSSHF** | **µ_ij_(x_ij_(t))_Low_** | **µ_ij_(x_ij_(t))_M_** | **µ_ij_(x_ij_(t))_H_** |
| --- | --- | --- | --- | --- |
| 0 | 1 | 0.8 | 0.2 |  |
| 0.25 | 2.15417 | 0.569166 | 0.430834 |  |
| 0.5 | 3.19773 | 0.360454 | 0.639546 |  |
| 0.75 | 4.14128 | 0.171744 | 0.828256 |  |
| 1 | 4.9944 | 0.00112 | 0.99888 |  |
| 1.25 | 5.76577 | 0 | 0.846846 | 0.153154 |
| 1.5 | 6.46322 | 0 | 0.707356 | 0.292644 |
| 1.75 | 7.09383 | 0 | 0.581234 | 0.418766 |
| 2 | 7.664 | 0 | 0.4672 | 0.5328 |
| 2.25 | 8.17954 | 0 | 0.364092 | 0.635908 |
| 2.5 | 8.64566 | 0 | 0.270868 | 0.729132 |
| 2.75 | 9.06712 | 0 | 0.186576 | 0.813424 |
| 3 | 9.44819 | 0 | 0.110362 | 0.889638 |
| 3.25 | 9.79274 | 0 | 0.041452 | 0.958548 |
| 3.5 | 10.10427 | 0 | 0 | 1 |
| 3.75 | 10.38594 | 0 | 0 | 1 |
| 4 | 10.64062 | 0 | 0 | 1 |
| 4.25 | 10.8709 | 0 | 0 | 1 |
| 4.5 | 11.0791 | 0 | 0 | 1 |
| 4.75 | 11.26735 | 0 | 0 | 1 |
| 5 | 11.43757 | 0 | 0 | 1 |
| 5.25 | 11.59147 | 0 | 0 | 1 |
| 5.5 | 11.73062 | 0 | 0 | 1 |
| 5.75 | 11.85643 | 0 | 0 | 1 |
| 6 | 11.97019 | 0 | 0 | 1 |
| 6.25 | 12.07305 | 0 | 0 | 1 |
| 6.5 | 12.16605 | 0 | 0 | 1 |
| 6.75 | 12.25013 | 0 | 0 | 1 |
| 7 | 12.32616 | 0 | 0 | 1 |
| 7.25 | 12.39491 | 0 | 0 | 1 |
| 7.5 | 12.45706 | 0 | 0 | 1 |
| 7.75 | 12.51326 | 0 | 0 | 1 |
| 8 | 12.56407 | 0 | 0 | 1 |
| 8.25 | 12.61001 | 0 | 0 | 1 |
| 8.5 | 12.65156 | 0 | 0 | 1 |
| 8.75 | 12.68911 | 0 | 0 | 1 |
| 9 | 12.72307 | 0 | 0 | 1 |
| 9.25 | 12.75378 | 0 | 0 | 1 |
| 9.5 | 12.78154 | 0 | 0 | 1 |
| 9.75 | 12.80664 | 0 | 0 | 1 |
| 10 | 12.82934 | 0 | 0 | 1 |
| 10.25 | 12.84986 | 0 | 0 | 1 |
| 10.5 | 12.86842 | 0 | 0 | 1 |
| 10.75 | 12.88519 | 0 | 0 | 1 |
| 11 | 12.90036 | 0 | 0 | 1 |
| 11.25 | 12.91408 | 0 | 0 | 1 |
| 11.5 | 12.92648 | 0 | 0 | 1 |
| 11.75 | 12.93769 | 0 | 0 | 1 |
| 12 | 12.94783 | 0 | 0 | 1 |
| 12.25 | 12.957 | 0 | 0 | 1 |
| 12.5 | 12.96528 | 0 | 0 | 1 |
| 12.75 | 12.97278 | 0 | 0 | 1 |
| 13 | 12.97955 | 0 | 0 | 1 |
| 13.25 | 12.98568 | 0 | 0 | 1 |
| 13.5 | 12.99122 | 0 | 0 | 1 |
| 13.75 | 12.99623 | 0 | 0 | 1 |
| 14 | 13.00076 | 0 | 0 | 1 |
| 14.25 | 13.00485 | 0 | 0 | 1 |
| 14.5 | 13.00855 | 0 | 0 | 1 |
| 14.75 | 13.0119 | 0 | 0 | 1 |
| 15 | 13.01493 | 0 | 0 | 1 |
| 15.25 | 13.01766 | 0 | 0 | 1 |
| 15.5 | 13.02013 | 0 | 0 | 1 |
| 15.75 | 13.02237 | 0 | 0 | 1 |
| 16 | 13.02439 | 0 | 0 | 1 |
| 16.25 | 13.02622 | 0 | 0 | 1 |
| 16.5 | 13.02788 | 0 | 0 | 1 |
| 16.75 | 13.02937 | 0 | 0 | 1 |
| 17 | 13.03072 | 0 | 0 | 1 |
| 17.25 | 13.03195 | 0 | 0 | 1 |
| 17.5 | 13.03305 | 0 | 0 | 1 |
| 17.75 | 13.03405 | 0 | 0 | 1 |
| 18 | 13.03496 | 0 | 0 | 1 |
| 18.25 | 13.03577 | 0 | 0 | 1 |
| 18.5 | 13.03651 | 0 | 0 | 1 |
| 18.75 | 13.03718 | 0 | 0 | 1 |
| 19 | 13.03778 | 0 | 0 | 1 |
| 19.25 | 13.03833 | 0 | 0 | 1 |
| 19.5 | 13.03882 | 0 | 0 | 1 |
| 19.75 | 13.03927 | 0 | 0 | 1 |
| 20 | 13.03967 | 0 | 0 | 1 |
